# Pre-implantation genetic testing for aneuploidy: motivations, concerns, and perceptions in a UK population

**DOI:** 10.1007/s10815-021-02130-3

**Published:** 2021-03-11

**Authors:** Benjamin P. Jones, Timothy Bracewell-Milnes, Lorraine Kasaven, Ariadne L’Heveder, Megan Spearman, Diana Marcus, Maria Jalmbrant, Joy Green, Rabi Odia, Srdjan Saso, Paul Serhal, Jara Ben Nagi

**Affiliations:** 1 Hammersmith Hospital, Imperial College NHS Trust, London, London W12 0HS UK; 2 Department of Surgery and Cancer, Imperial College London, Du Cane Road, London, W12 0NN UK; 3 Centre for Reproductive and Genetic Health, Great Portland Street, London, W1W 5QS UK

**Keywords:** Infertility, In vitro fertilisation, IVF, Perceptions, PGT-A, Pre-implantation genetic testing for aneuploidies

## Abstract

**Purpose:**

Pre-implantation genetic testing for aneuploidies (PGT-A) is a technique used as part of in vitro fertilisation to improve outcomes. Despite the upward trend in women utilising PGT-A, data on women’s motivations and concerns toward using the technology, and perceptions having undergone the process, remain scarce.

**Methods:**

This cross-sectional survey, based at a fertility clinic in the UK, utilised an electronic questionnaire to assess the motivations of women who undergo PGT-A and their perceptions and attitudes toward PGT-A after using it.

**Results:**

One hundred sixty-one women responded. The most significant motivating factors to undergo PGT-A were to improve the probability of having a baby per cycle (9.0 ± 2.1) and enhance the chance of implantation (8.8 ± 2.5). The least important motivations were reducing the number of embryos transferred per cycle (2.7 ± 3.3) and saving money by reducing the number of procedures required (4.6 ± 3.4). The most significant concerning factors identified included not having embryos to transfer (5.7 ± 3.4) and the potential for embryo damage (5.2 ± 3.3). The least concerning factors included religious (0.6 ± 1.7) or moral (1 ± 2.2) concerns. The majority of women were satisfied/very satisfied following treatment (*n* = 109; 68%). The proportion of those who were satisfied/very satisfied increased to 94.2% (*n* = 81) following a successful outcome, and reduced to 43.5% (*n* = 27) in those who had an unsuccessful outcome or had not undergone embryo transfer (*p* < 0.001).

**Conclusion:**

This study highlights that perceptions amongst women who use PGT-A are mostly positive. We also demonstrate a significant association between satisfaction and reproductive outcomes, with those who achieve a live birth reporting more positive perceptions toward PGT-A.

**Supplementary Information:**

The online version contains supplementary material available at 10.1007/s10815-021-02130-3.

## Introduction

Pre-implantation genetic testing for aneuploidies (PGT-A) is a technique used as part of in vitro fertilisation (IVF) to improve reproductive outcomes [1–3]. The main reason for suboptimal reproductive outcomes in women of advanced maternal age (AMA), or those with recurrent miscarriages (RM) or repetitive implantation failure (RIF) is due to embryonic aneuploidy [4–6]. PGT-A facilitates embryo selection by allowing the opportunity to prioritise chromosomally normal embryos for transfer to the uterus. This technique has been suggested to be superior to traditional methods of embryo selection utilising morphology alone, which is considered to be an inadequate predictor of chromosomal abnormalities [7].

Following various technical advances in PGT-A, in particular the implementation of comprehensive chromosome screening, the transfer of euploid embryos has been shown to result in higher implantation, ongoing pregnancy, and delivery rates with reduced pregnancy loss compared to unscreened embryos [8]. This mitigates the negative clinical impact chromosomal aneuploidy has upon reproductive outcomes. In addition, with live birth rates similar to that of undertaking double embryo transfer (ET) with unscreened embryos, the use of PGT-A facilitates the implementation of single ET policies and as such helps avoid the complications associated with multiple gestation [9].

Despite the potential advantages offered by PGT-A, current evidence is not only limited in quantity but comprehensive systematic review of outcomes also are difficult owing to statistical heterogeneity and methodological diversity, particularly between different CCS techniques and stage of biopsy. However, whereas superior outcomes have also been identified in women with AMA [7], RM [1], and RIF [3], there remains an absence of high-quality randomised controlled trial (RCT) data. In women with good prognoses, whilst initial RCTs identified an improvement in implantation, pregnancy, and live birth rates [2], more recent data has failed to show improvement [10], highlighting the ongoing controversary regarding its widespread implementation. This lack of robust, high-quality evidence has led to a recent Cochrane review being unable to recommend the routine use of PGT-A in clinical practice [11]. Moreover, the Human Fertilisation and Embryology Authority (HFEA) recommendation for the use of PGT-A as an *add on*, remains *red* for blastocyst stage biopsies, because of the lack of evidence that it improves live birth rates.

In addition, there remain some reservations amongst women regarding its use. There have been concerns about the effect of the biopsy on the embryo [12], although evolution in technique and improved culture methods enables a later biopsy, which is less harmful [13]. Other potential concerns for the use of PGT-A include the risk of no euploid embryos subsequently being available for transfer, and the additional associated financial expense. Moreover, lack of standardisation between genetic techniques used and categorisation of what constitutes normality may impact confidence in the process.

Despite the upward trend in women utilising PGT-A [14], and the growing body of literature investigating its efficacy and safety, there is little data about women’s motivations and concerns toward using the technology, and perceptions having undergone the process [15]. The objective of this study was to investigate the motivations of women who undergo PGT-A and their perceptions and attitudes toward it after undergoing PGT-A as part of IVF treatment.

## Materials and methods

An electronic questionnaire was sent to all the women who had consented to be contacted for research purposes after undergoing IVF with PGT-A at the Centre for Reproductive and Genetic Health (CRGH) in London over a 5-year period between 1 January 2014 and 31 December 2018. It consisted of 37 questions (Supplemental Appendix 1) and was distributed via e-mail through SurveyMonkey between 8 October 2019 and 31 January 2020.

An online questionnaire, utilising a 10-point Likert scale, was created to assess motivations and concerns toward PGT-A, as well as outcomes and perceptions following IVF using PGT-A. Motivating and concerning factors were quantified out of 10 depending on perceived significance (0 = insignificant; 10 = very significant). A mixture of closed and Likert-scaled questions was used to assess perceptions. Following the initial invitation, two further reminder e-mails were sent if the questionnaire was not completed.

### Details of ethics approval

The IVF process and use of PGT-A were explained comprehensively by a select group of fertility specialists, and written consent was received. Local institutional review board approval (IRB-0001C) was obtained on 7 October 2019 to undertake the retrospective electronic questionnaire. At the time of their treatment, all women consented to be contacted in the future for research purposes.

### Clinical protocols

All women underwent controlled ovarian stimulation with either mid-luteal phase agonist or antagonist protocols utilising gonadotrophins individualised according to previous history, body mass index, age, and baseline markers of ovarian reserve including anti-Mullerian hormone and antral follicle count. Oocyte maturation was triggered from day 10 of the cycle onwards following the identification of three or more follicles > 17 mm in diameter on ultrasound. Transvaginal oocyte retrieval was undertaken 37 h later under ultrasound guidance.

Oocytes were fertilised with IVF or intracytoplasmic sperm injection (ICSI). Until 7 October 2015, fertilised oocytes were cultured in pre-equilibrated sequential SAGE medium (Origio, Denmark), whilst after this date, pre-equilibrated SAGE 1-step medium (Origio, Denmark) was used. Embryos were subsequently cultured in either a benchtop incubator or a time-lapse incubator (EmbryoScope, Vitrolife, Denmark).

All embryos underwent assisted hatching on day 3 post injection. Embryos that formed blastocysts on day 5 or 6 post insemination (via IVF or ICSI) were subjected to embryo biopsy and genetic analysis. In our unit, comparative genomic hybridisation was used prior to 2017, and next-generation sequencing was used in those who presented from 2017 onwards, which have been described in detail previously [16]. Following biopsy, embryos were incubated for 5–10 min to allow complete cavity collapse prior to vitrification. The blastocysts were then individually cryopreserved using Cook vitrification kits (Cook Medical, Sydney) for washing and dehydration before loading into the Cryolock (Biotech Inc., Ireland) and submerged into liquid nitrogen.

Warming was carried out at 37 °C on the day of embryo transfer where the Cryolock was rapidly removed from the liquid nitrogen and immediately immersed into Cook warming solutions (Cook Medical, Sydney). The blastocysts were subsequently transferred to the pre-equilibrated culture-dishes containing SAGE media and incubated to allow blastocyst recovery and blastocoel cavity re-expansion. Warmed blastocysts were then assessed after 2 to 3 h for viability and re-expansion prior to embryo transfer.

Couples who had a euploid embryo to transfer subsequently underwent vitrified warmed single embryo transfer cycles as described previously in detail [17]. Suitable blastocysts were warmed on the day of embryo transfer. All couples underwent single embryo transfer.

### Statistical analyses

SPSS version 24 software (SPSS, Chicago, IL, USA) was used for analysis. Descriptive statistical analysis was described as mean ± standard deviation or median ± range. The individualised probability of experiencing miscarriage or live birth was estimated according to indication for PGT-A, according to published literature. The estimated live birth rates following transfer of a single euploid embryo in women with AMA, RM, and RIF was 52.9% [5], 52.4% [18], and 62.5%, respectively [18]. When considering the individualised estimation of the probability of miscarriage per pregnancy following PGT-A for AMA, RM, and RIF, rates of 2.7% [5], 14.3% [18], and 11.8% [18] were respectively used. Chi-squared test was used to assess for associations between perceptions and reproductive outcomes. Statistical significance was set at *P* < 0.001.

## Results

The survey was sent to 333 women who underwent IVF with PGT-A during the study period. The response rate was 48.3% (*n* = 161). One hundred fifty (93%) respondents completed it in its entirety whereas it was partially completed by 11 (7%). The cohort demographics are summarised in Table 1. The median age of participants was 38 years (range 19–46) and the median BMI was 22.1 (range 17.6–42). Almost three quarters (*n* = 120; 74.5%) of the cohort were married at the time of undergoing PGT-A and the most preponderant ethnicity was white British (*n* = 121; 75.2%). The majority were nulliparous (*n* = 116; 72%), had a higher education degree (*n* = 145; 90%), and were either self-employed or in full time employment (*n* = 126; 78%). Whilst some had more than one indication, the most prevalent indication for undergoing PGT-A was AMA (*n* = 84; 35.1%). Other reasons included RIF (*n* = 77; 32.2%), defined herein as ≥ 3 unsuccessful embryo transfers, despite the use of good-quality embryos [19], RM (*n* = 42; 17.6%), previous pregnancy affected by chromosomal abnormality (*n* = 22; 9.2%), and personal request (*n* = 14; 5.9%).

**Table 1:** Cohort demographics

		Number	%
Age	< 25	1	0.6
	25–29	4	2.5
	30–34	28	17.4
	35–39	88	54.7
	40–44	39	24.2
	≥ 45	1	0.6
Ethnicity	White	128	79.5
	Asian	15	9.3
	Black	4	2.5
	Mixed	3	1.9
	Middle Eastern	6	3.7
	Other	5	3.1
Relationship status	Single	6	3.7
	In relationship	35	21.8
	Married	120	74.5
Employment	Employed (full time)	106	65.8
	Employed (part time)	26	16.1
	Self-employed	20	12.4
	Student	1	0.6
	Housewife	5	3.1
	Other	3	1.9
Education	GCSEs	3	1.9
	A levels/diploma	13	8.1
	Undergraduate degree	56	24.8
	Postgraduate degree	69	42.9
	Doctorate	20	12.4

The motivations and concerns expressed by participants regarding PGT-A are summarised in Tables 2 and 3. More than three quarters of the respondents felt that improving the chances of the embryo implanting (*n* = 125; 77.6%) and improving the chances of having a baby by IVF (*n* = 126; 78.3%) were “very significant” (score 9–10). One hundred fourteen women (70.8%) perceived the improvement in probability of having a healthy baby had a very significant (score 9–10) influence on their decision and 112 (69.6%) thought similarly toward the reduction in risk of miscarriage. Lower proportions deemed that reducing the chance of having a baby with birth defects (*n* = 89; 55.3%), reducing the time to achieve pregnancy (*n* = 71; 44.1%), and decreasing the risk of needing to have a termination of pregnancy (*n* = 70; 43.5%) were very significant (score 9–10). As demonstrated in Table 2, the most significant motivating factors to undergo PGT-A were to improve the probability of having a baby per cycle (9.0 ± 2.1), enhance the chance of implantation (8.8 ± 2.5), and improve the probability of having a healthy baby (8.8 ± 2.3). The least important motivating factors were reducing the number of embryos transferred per cycle to reduce the probability of twins (2.7 ± 3.3), saving money by reducing the number of procedures required (4.6 ± 3.4), and reducing the number of ETs performed (5.4 ± 3.8).

**Table 2:** Motivations for undergoing PGT-A

Motivation	Mean	SD
“Improve my chances of having a baby per IVF cycle overall”	9.0	2.1
“Improve the chance of having a healthy baby”	8.8	2.3
“Improve the chance of the embryo implanting”	8.8	2.5
“Reduce the risk of miscarriage”	8.5	2.7
“Reduce the chance of having a baby with birth defects”	7.8	3.0
“Reduce the amount of time it takes to get pregnant”	7.2	3.1
“Reduce the risk of needing to have a termination of pregnancy”	6.6	3.7
“Reduce the number of embryo transfer procedures performed”	5.4	3.8
“Save money by reducing the number of fertility procedures I needed”	4.6	3.4
“Reduce the numbers of embryos transferred per transfer, so reducing my chances of having twins”	2.7	3.3

**Table 3:** Concerns regarding PGT-A

Concern	Mean	SD
“PGS could result in me not having any embryos to transfer”	5.7	3.4
“PGS damaging my embryos”	5.2	3.3
“The cost of PGS”	5.1	3.3
“I will not have any or enough embryos to do PGS”	4.8	3.7
“PGS treatment not improving my pregnancy rates per IVF cycle”	4.6	3.5
“Concern the technology will give an incorrect genetic result of my embryos”	4.4	3.4
“PGS may yield mosaic embryos that will potentially be discarded”	4.3	3.6
“PGS treatment increases the amount of time before having an embryo transfer”	3.4	2.9
“Requiring multiple stimulation of my ovaries to generate sufficient embryos to perform PGS”	2.8	3.2
“Concern for discarding surplus genetically abnormal embryos”	2.8	3.2
“PGS treatment would cause stress for my partner/family”	2.2	2.7
“Missing many days of work”	1.9	2.9
“My local fertility unit not offering PGS so having to change fertility clinics”	1.2	2.5
“Using PGS to select genetically normal embryos goes against my moral beliefs”	1.0	2.2
“Using PGS to select genetically normal embryos goes against my religious beliefs”	0.6	1.7

The participants’ perceptions to potential concerns regarding their decision to undergo PGT-A are summarised in Table 3. The most significant concerning factors identified were that PGT-A could result in not having any embryos to transfer (5.7 ± 3.4), the potential for damage to be caused to embryos (5.2 ± 3.3), and the associated financial costs involved (5.1 ± 3.3). The least concerning factors included PGT-A going against religious (0.6 ± 1.7) or moral (1 ± 2.2) beliefs, and having to change clinics if their local clinic did not offer PGT-A (1.2 ± 2.5). More than half of the cohort perceived the potential need for multiple cycles to attain a suitable embryo for PGT-A (*n* = 82, 50.9%) as being very insignificant (score 0–1). Moreover, more than half of the cohort perceived the potential for treatment to result in missing days off work (*n* = 105, 65.2%) and cause stress for partners and family (*n* = 87, 54.1%) as being very insignificant (score 0–1).

**Fig. 1:**
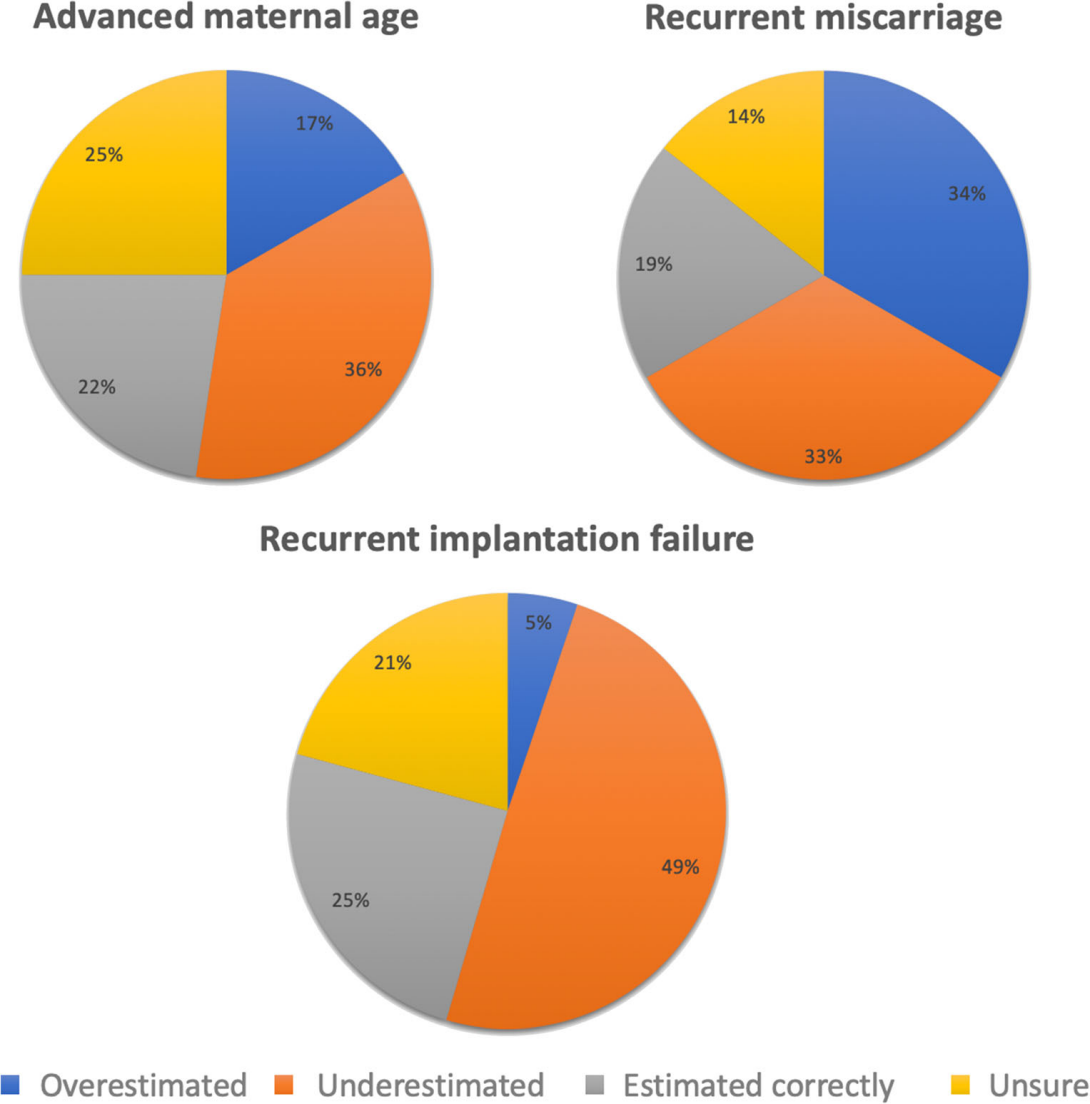
Perceived probability of live birth success following embryo transfer with a euploid embryo, stratified by indication for undergoing PGT-A

With regard to the individualised probability of achieving a live birth per ET following PGT-A, 13 (8.1%) women believed it to be < 20%, whereas 30 (20%) thought it was between 20 and 40%. Thirty-five women (21.7%) perceived the probability of success to be 40–60%, a further 35 (21.7%) thought 60–80%, and ten (6.2%) believed it to be 80–100%. A fifth (*n* = 35; 21.7%) of respondents were unsure of the likelihood of success per euploid ET. Following calculation using the live birth rate following PGT-A in the context of RM, where the live birth rate per transfer has been shown to be 52.4% [18], just 19% (*n* = 8) of those who underwent PGT-A for this indication estimated the probability correctly. Fourteen (33.3%) women overestimated their chance of success, 14 (33.3%) underestimated the probability, and six (14.3%) were unsure. In those who used PGT-A because of previous RIF, 19 (24.7%) participants correctly estimated their probability of success per ET, which has been shown to be 62.5% [18], whereas four (5.2%) overestimated, 38 (49.4%) underestimated, and 16 (20.8%) were unsure. In those who utilised PGT-A because of AMA, 19 (22.6%) women correctly estimated their probability of live birth, which has been shown to be 52.9% [5]. However, 14 (16.7%) women overestimated the chances of success, 30 (35.7%) underestimated, and 21 (25%) were unsure. The perceived probability of live birth success following embryo transfer with a euploid embryo, stratified by indication for undergoing PGT-A, is represented in Fig. 1.

**Fig. 2:**
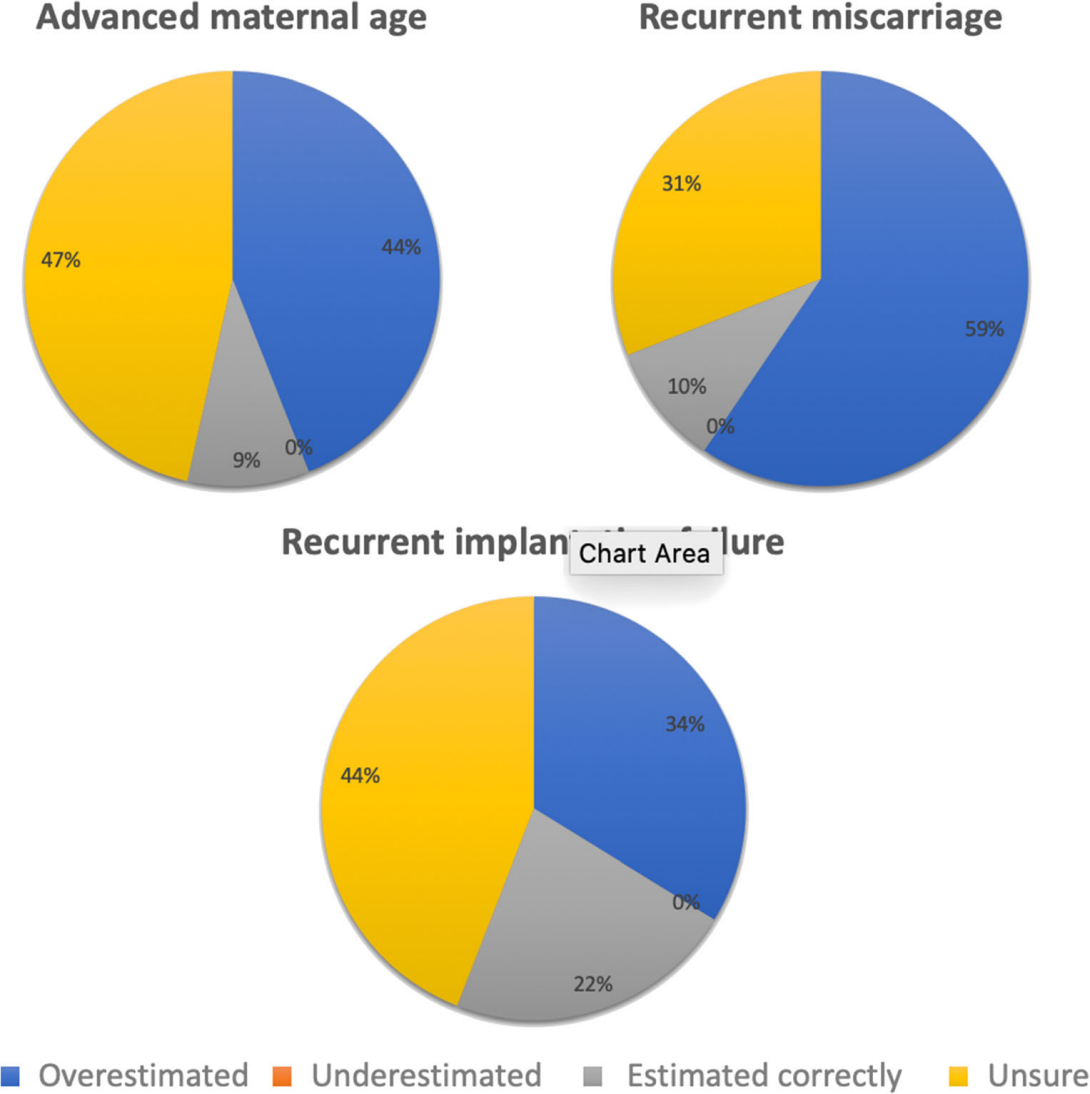
Perceived probability of miscarriage following pregnancy with a euploid embryo, stratified by indication for undergoing PGT-A

With regard to miscarriage, 28 (17.4%) women believed the chance of miscarriage following pregnancy to be 0–20%, whereas 33 (20.5%) thought it was between 20 and 40%. Eighteen women (11.2%) perceived the probability to be 40–60%, whereas a further 6 (3.7%) and 7 (4.3%) believed it to be 60–80% and 80–100%, respectively. Sixty-six (41.0%) of respondents were unsure of the likelihood of miscarriage following pregnancy. When considering the individualised estimation of the probability of miscarriage per pregnancy following PGT-A, in those who underwent treatment for RM, four (9.5%) correctly estimated the probability of miscarriage per pregnancy, which has been shown to be 14.3% [18], whereas 25 (59.5%) overestimated the risk. In those with RIF, 17 (22.1%) correctly predicted the chance of miscarriage per pregnancy of 11.8% [18], whereas 26 (33.8%) overestimated the risk. In those who underwent PGT-A for AMA, eight (9.5%) correctly estimated their chance of miscarriage per cycle, which has been demonstrated to be 2.7% [5], and 37 (44%) overestimated the risk. No women underestimated the chance of miscarriage across any of the groups. The perceived probability of miscarriage following embryo transfer with a euploid embryo, stratified by indication for undergoing PGT-A, is represented in Fig. 2.

The majority of women (*n* = 98; 60.9%) understood that PGT-A would not guarantee a live birth free from chromosomal abnormalities. 50.3% (*n* = 81) stated that if their fertility treatment was successful, they would inform their children of their IVF treatment with PGT-A and the majority (*n* = 115; 71.4%) felt well informed should they become pregnant; routine pre-natal screening in pregnancy is still recommended to confirm the PGT-A result of a genetically normal embryo.

**Fig. 3:**
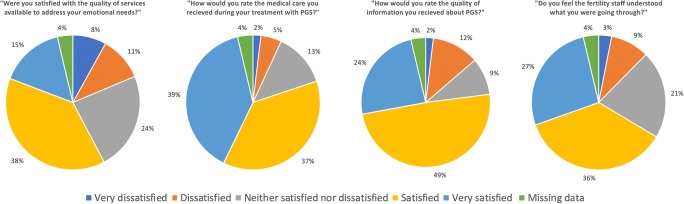
Perceptions and experiences of the women following IVF with PGT-A

At the time of undertaking the questionnaire, the majority of women (*n* = 112; 71.8%) had undergone ET with the embryos which had undergone PGT-A, whereas 44 (28.2%) had not yet. The majority of women required two or less cycles to create at least one euploid embryo (*n* = 115; 71.5%). Seven women (4.3%) did not have a suitable euploid embryo for transfer following PGT-A. With regard to reproductive outcomes, from the 112 women who subsequently underwent ET, 98 (87.5%) resulted in positive pregnancy tests whereas 12.5% (*n* = 14) were unsuccessful. Of those who achieved pregnancy following PGT-A, outcomes were available from 94 women. Of those, live birth was subsequently achieved in 84 (89.4%) women and seven (7.4%) were pregnant at the time of the survey. Five (8.0%) women miscarried, and data from four (4.1%) women was unavailable. With regard to their personal experience of PGT-A, the reported experiences of the women in the cohort are summarised in Fig. 3.

The majority of women were either satisfied or very satisfied following IVF treatment with PGT-A (*n* = 109; 68%) whereas 15% were dissatisfied or very dissatisfied. The proportion of those who were satisfied or very satisfied increased to 94.2% (*n* = 81) in those who had a successful outcome, defined as either having achieved a live birth or being pregnant, whereas it was 43.5% (*n* = 27) in those who had an unsuccessful outcome (miscarriage or unsuccessful ET), or whom had not yet undergone ET. The association between satisfaction with treatment and reproductive outcome was statistically significant (*p* < 0.001). Two-thirds of women (*n* = 108; 67.1%) would utilise PGT-A as part of IVF treatment again in the future, and three quarters (*n* = 123; 76.4%) would recommend it to a friend or family member. In those women who achieved a live birth or were currently pregnant (*n* = 91), 92.3% (*n* = 84) would recommend to a friend or family member, six (6.6%) were unsure and one (1.1%) would not. Similarly, in those who were pregnant or achieved live birth, 84.6% (*n* = 77) would use PGT-A again, eight (8.8%) were unsure, and six (6.6%) would not. In contrast, in those who miscarried (*n* = 5), 40% (*n* = 2) would use PGT-A in future cycles and recommend to family or friends. In those who underwent ET but were unsuccessful (*n* = 14), 33.3% (*n* = 4) would use PGT-A in the future and 58.3% (*n* = 7) would recommend to family and friends. In those who had undergone PGT-A who have not yet undergone ET, 47.9% (*n* = 23) would use it again in the future and 58.3% (*n* = 7) would recommend to family and friends. When comparing between those who had a successful reproductive outcome with those who had an unsuccessful outcome or whom had not undergone ET, there were significant associations between outcomes and both desire to use PGT-A in the future (*p* < 0.001), and whether they would recommend to family and friends (*p* < 0.001).

## Discussion

The data presented herein highlights that the majority of women undergoing PGT-A as part of IVF have a positive experience, although satisfaction is significantly related to subsequent reproductive outcomes. This study provides a perspective into the motivations and concerns women feel regarding the process of PGT-A as well as their perceptions regarding success rates of PGT-A.

The findings demonstrated herein are supported by a recent cross-sectional survey on decision-making in the use of PGT-A, which identified the most prevalent motivation to utilise PGT-A was to have a healthy baby, which was reported by 56% of their cohort [12]. Whereas the use of PGT-A minimises the risk of genetic abnormalities secondary to aneuploidy, it is essential women using PGT-A understand the aetiology of congenital and structural abnormalities are multifactorial and can arise due to a number of other genetic and non-genetic factors. Despite being heavily motivated by the aim of having a healthy baby, the majority of women (60.9%) in our cohort understood PGT-A would not guarantee a live birth free from chromosomal abnormalities. Conversely, another recent study which investigated predictors of decision regret and anxiety following PGT-A found that the most common reason for a woman to choose PGT-A was to “improve the efficiency of IVF and have a baby sooner” [15]. Whilst this was not observed in our study, reducing the time to achieve live birth was also important, being viewed as a significant motivator in 44.1% of cases.

The association between pregnancy loss and psychological morbidity is well established, as 8–20% experience symptoms above the threshold for moderate depression and 18–32% likewise for anxiety, between 4 to 6 weeks after pregnancy loss. Women with a history of infertility in particular, as is the case in this cohort, have been found to be at increased risk of psychological morbidity following miscarriage [20]. It is therefore unsurprising that more than two-thirds of this cohort perceived the reduction in risk of miscarriage as a very significant motivation to use PGT-A.

Interestingly, the women in our cohort overestimated the risk of miscarriage in IVF cycles using PGT-A with 59.5%, 33.8%, and 44% overestimating their risk of miscarriage for PGT-A in women with RM, RIF, and those of AMA, respectively. This is important as reducing miscarriage risk is a key benefit gained from the use of PGT-A. A retrospective cohort study in women with AMA has demonstrated a reduction in miscarriage rates between those who used PGT-A and those who did not (10.7% vs 38.1%) [7]. This reduction in miscarriage rates not only reduces the psychological morbidity associated with miscarriage but also avoids the stress, financial expense, and heartbreak of having to undergo further treatment which may not be successful. Whilst managing expectations is important, the perceived higher risk of miscarriage could add additional emotional strain during what is already a stressful process, and as such more reassuring counselling may reduce stress following successful ETs. Moreover, the majority of this cohort underestimated their chances of achieving a live birth, which could further potentiate anxiety during the IVF process. Contrastingly, however, almost 20% (*n* = 32) of the cohort overestimated their chances of success which could lead to unnecessary disappointment secondary to unrealistic expectations. This highlights the need for more extensive counselling regarding PGT-A and likelihood of achieving live birth and miscarriage per ET.

Regarding concerns toward PGT-A, the most significant ones identified herein result were the eventuality that they may not have any embryos for transfer, potential damage to embryos during the biopsy process, and the associated financial costs. There remains limited evidence of women’s concerns regarding the use of PGT-A in published literature. One study found that 39% expressed some degree of regret [15], and multiple regression analysis demonstrated an inverse relationship between embryo ploidy and decision regret, with lower numbers of euploid embryos available for transfer being associated with a greater degree of regret. This supports our data highlighting the main concern of not having a sufficient number of embryos for transfer. Similarly, a cross-sectional survey on decision-making regarding the use of PGT-A found that the most common reason for not using PGT-A was to avoid the scenario of having no embryos available for transfer [12]. Whereas it is understandable distress may be caused by undergoing IVF without creating any euploid embryos, the alternative situation entails the transfer of a morphologically normal aneuploid embryo which does not implant or result in miscarriage. Notably, a study analysing reasons why couples stop IVF treatment found the most common reason to be stress (39%), with the two main stressors identified as the negative impact on the couple’s relationship and being too depressed or anxious to continue [21]. By improving reproductive outcomes, PGT-A may offer an opportunity to reduce stress by enhancing reproductive outcomes.

In support of the findings demonstrated herein, the financial cost of PGT-A has previously been identified as a reason why woman may choose not to use PGT-A, with 31% of patients choosing not to use PGT-A as they perceived cost reduction as a key priority [12]. Whilst PGT-A adds additional expense to an already costly process, it remains unclear whether reducing the number of ETs reduces overall expenditure, as data from economic analyses remain inconclusive. A recent study in nearly 9000 women demonstrated an overall cost saving for women with greater than one embryo who chose to undergo PGT-A, as opposed to IVF alone [22]. On the other hand, a multicentre RCT using PGT-A in AMA patients identified that the cost per baby was found to be 8% higher in the PGT-A group [5]. However, it has been suggested that the use of next-generation sequencing would lower this cost by as much as 12% per baby [23]. Whilst our findings identify cost as one of the more significant concerns, other studies have not found it to be as important in the decision-making process. A study on regret following PGT-A did not find any difference in decision regret when comparing those paying personally, to those covered by insurance [15], and another study investigating reasons why women discontinue IVF treatment noted that a significant number of patients terminate treatment of their own volition and not because of financial reasons [21].

Concerns regarding harm to embryo during the biopsy process have been previously cited as reason for not pursing PGT-A [12]. There is evidence to suggest that the process of embryo biopsy may be harmful, with one study demonstrating a 39% reduction in implantation rates with cleavage-stage biopsy compared with non-biopsied day 3 embryos [24]. However, evolution of PGT-A techniques has resulted in improved culture methods allowing for a later and less harmful trophectoderm biopsy [13]. There is also the potential prospect for non-invasive PGT-A in the future which may negate the need for biopsy altogether.

Overall the respondents felt positive about their PGT-A experience with more than two-thirds stating they would use it in future fertility treatment and more than three quarters agreeing they would recommend to a friend or family member. This is similar to another study whereby 94% of all respondents reported satisfaction with their decision to pursue PGT-A, with the vast majority of women concluding that, regardless of outcome, the information obtained during the PGT-A process would be valuable for future reproductive planning [15]. Unsurprisingly, our data highlights that perceptions are more positive in those who achieved a live birth or were pregnant at the time of undertaking the questionnaire, and more negative in those who miscarried or underwent unsuccessful ET. This is reaffirmed by a previous study which identified that women who conceived following euploid transfer reported less regret than those who miscarried or failed to conceive [15].

This study is one of very few studies to investigate women’s perceptions on PGT-A. Whilst there was a reasonable response rate relative to previous studies, there is an unavoidable element of bias introduced in any questionnaire. Moreover, the socioeconomic background of the participants was predominantly white, highly educated, and employed, which is not reflective of the entire population. Furthermore, as the survey was cross-sectional in nature, the timing of completion will have varied between participants, and further bias may be encountered by subsequent outcomes such as achievement of a live birth, as epitomised by the results highlighting better perceptions in this group. Additionally, the survey did not allow respondents to document additional factors influencing their decision-making and it is possible the important factors may not have been addressed. As such, a larger, prospective study should be undertaken, using a cohort more representative of the general population, before the women know their outcomes, to help overcome the limitations present in this study.

## Conclusion

IVF requires an immense emotional, physical, and financial commitment which is not undertaken lightly by women. Poor outcomes will inevitably be associated with significant psychological morbidity with evidence of treatment failure. Rather than facing the physical, psychosocial, and economic burden associated with miscarriage, an increased number of ETs, and a longer time to achieve pregnancy, PGT-A offers an opportunity to enhance reproductive outcomes. Whilst PGT-A is viewed positively in the data presented herein, and in other studies as discussed, perceptions are lower for those with unsuccessful outcomes. Moreover, perceptions of estimated miscarriage and live birth probabilities remain inaccurate, as highlighted in this study. This highlights an important role for extensive reproductive counselling and support for women choosing to pursue PGT-A.

## Supplementary Information

ESM 1 (DOCX 42 kb)

## Data Availability

Data are available on request from authors.
