# Supplementary material for: Pre-implantation genetic testing for aneuploidy: motivations, concerns, and perceptions in a UK population
Source: J Assist Reprod Genet. 2021 Mar 11;38(8):1987–96. doi: 10.1007/s10815-021-02130-3 (PMC8417165; doi:10.1007/s10815-021-02130-3)
Supplement: Supplementary file 1 — (DOCX 42 kb) [file 10815_2021_2130_MOESM1_ESM.docx]

**Pre-implantation genetic screening (PGS) survey**

**Section 1: Demographic Information**

1. **What is your ethnicity?**

**White:**

White British  White Irish  Other

**Mixed Ethnicity:**

White/Black Caribbean  White/Black African  White/Asian

Other

**Asian/Asian British:**

Indian  Pakistani  Bangladeshi  Chinese

Other

**Black/African/Caribbean/Black British:**

African  Caribbean

Other

**Other ethnic group:**

Arab  Other

1. **What was your relationship status at the time you embarked on PGS?**

Married  In a relationship (unmarried)  Single

1. **How many children did you have prior to embarking on PGS?**

None  One  Two  Three  More than three

1. **What was your employment status at the time you chose to embark on PGS?**

Employed (Full time)  Employed (Part time)  Self Employed

Student  Housewife  Unemployed

Other

1. **What is your educational level?**

No formal qualifications  GCSEs  A-Levels/Diploma

University degree  Post-graduate degree

Other

**Section 2: Motivations and Concerns Regarding PGS**

1. **(i) When making the decision to have PGS done, how important was it for you to……? [Please grade the following motivations from 0 to 10 according to their significance to you, where 0 = insignificant and 10 = very significant.]**
   1. Improve the chance of the embryo implanting

0 1 2 3 4 5 6 7 8 9 10

- 1. Reduce the risk of miscarriage

0 1 2 3 4 5 6 7 8 9 10

- 1. Improve the chance of having a healthy baby

0 1 2 3 4 5 6 7 8 9 10

- 1. Reduce the chance of having a baby with birth defects

0 1 2 3 4 5 6 7 8 9 10

- 1. Reduce the risk of needing to have a termination of pregnancy

0 1 2 3 4 5 6 7 8 9 10

- 1. Reduce the numbers of embryos transferred per transfer, so reducing my chances of having twins

0 1 2 3 4 5 6 7 8 9 10

- 1. To reduce the number of embryo transfer procedures performed

1. 1 2 3 4 5 6 7 8 9 10
   1. Save money by reducing the number of fertility procedures I needed

0 1 2 3 4 5 6 7 8 9 10

- 1. Reduce the amount of time it takes to get pregnant

1. 1 2 3 4 5 6 7 8 9 10
   1. Improve my chances of having a baby per IVF cycle overall
2. 1 2 3 4 5 6 7 8 9 10

**7) What were your concerns regarding PGS?**

**[Please grade the following concerns from 0 to 10 according to their significance to you, where 0 = insignificant and 10 = very significant.]**

1. PGS could result in me not having any embryos to transfer

0 1 2 3 4 5 6 7 8 9 10

1. PGS damaging my embryos

0 1 2 3 4 5 6 7 8 9 10

1. Concern the technology will give an incorrect genetic result of my embryos

0 1 2 3 4 5 6 7 8 9 10

1. I will not have any or enough embryos to do PGS

0 1 2 3 4 5 6 7 8 9 10

1. The cost of PGS

0 1 2 3 4 5 6 7 8 9 10

1. My local fertility unit not offering PGS so having to change fertility clinics

0 1 2 3 4 5 6 7 8 9 10

1. Requiring multiple stimulations of my ovaries to generate sufficient embryos to perform PGS

0 1 2 3 4 5 6 7 8 9 10

1. Missing many days of work

0 1 2 3 4 5 6 7 8 9 10

1. PGS treatment increases the amount of time before having an embryo transfer

0 1 2 3 4 5 6 7 8 9 10

1. PGS treatment not improving my pregnancy rates per IVF cycle

0 1 2 3 4 5 6 7 8 9 10

1. PGS treatment would cause stress for my partner/family

0 1 2 3 4 5 6 7 8 9 10

1. Concern for discarding surplus genetically abnormal embryos

0 1 2 3 4 5 6 7 8 9 10

1. Using PGS to select genetically normal embryos goes against my religious beliefs

0 1 2 3 4 5 6 7 8 9 10

1. Using PGS to select genetically normal embryos goes against my moral beliefs

0 1 2 3 4 5 6 7 8 9 10

1. PGS may yield mosaic embryos that will potentially be discarded

0 1 2 3 4 5 6 7 8 9 10

**Section 3: Your experience of PGS**

**For each question, tick the response that is closest to YOUR CURRENT thoughts and feelings about your experience of your fertility treatment with PGS**

|  |  | **Very dissatisfied** | **Dissatisfied** | **Neither satisfied or dissatisfied** | **Satisfied** | **Very satisfied** |
| --- | --- | --- | --- | --- | --- | --- |
| **9** | **Were you satisfied with the quality of services available to you to address your emotional needs?** |  |  |  |  |  |
| **10** | **How would you rate the quality of information you received about PGS?** |  |  |  |  |  |
| **11** | **How would you rate the medical care you received during your treatment with PGS?** |  |  |  |  |  |
| **12** | **Do you feel the fertility staff understood what you were going through?** |  |  |  |  |  |
| **13** | **Were you satisfied with your interactions with fertility medical staff?** |  |  |  |  |  |
| **14** | **Overall, how would you rate your experience of IVF with PGS?** |  |  |  |  |  |

1. **Did you feel your best interests were always the primary concern of the medical staff during your fertility treatment?**

Yes  No  Unsure

1. **Now that you have gone through the process, do you feel that you were sufficiently knowledgeable to make the decision to use PGS?**

Yes  No  Unsure

1. **In the event PGS resulted in mosaic embryo(s), did you feel you had understanding of the implications of the results?**

Yes  No  Unsure  Not applicable

1. **In the event you had an embryo with mosaicism you requested to transfer, did you have access to genetic counselling?**

Yes  No  Unsure  Not applicable

1. **Did you understand the implications of transferring an embryo with mosaicism after you had received genetic counselling?**

Yes  No  Unsure  Not applicable

1. **Did you understand the potential benefits of PGS are currently inconclusive in terms of medical evidence?**

Yes  No  Unsure

1. **Would you participate in IVF treatment with PGS for future fertility treatment?**

Yes  No  Unsure

1. **Would you recommend IVF treatment with PGS to a friend or family member who needs fertility treatment?**

Yes  No  Unsure

**Section 4: Information and decision-making surrounding PGS**

1. **How did you first find out about PGS?**

Internet  Newspaper or magazine advert  Radio or TV advert

Social media  Family/friend  GP

Gynaecologist  Fertility clinic  IVF seminar

Don’t recall  Other (please specify)

1. **On a scale of 0-10 what were your first impressions of PGS? (0 = very bad idea, 10 = very good idea)**

0 1 2 3 4 5 6 7 8 9 10

1. **How long had you been trying to get pregnant before you heard about PGS?**

Less than 6 months

Between 6 months and 1 year

1 to 2 years

2 to 3 years

3 to 4 years

4 to 5 years

5 or more years

1. **How much research did you do into PGS before deciding to use it during your IVF cycle?**

More than 5 hours  1-5 hours  Less than 1 hour  None

1. **Who do you feel had the most influence on you making the decision to use PGS (please tick one box only)**

Partner  Family  Friends

Fertility provider  Obstetrician and gynaecologist  GP

1. **How useful did you find the information you received surrounding PGS from your fertility clinic? (0= not useful, 10= very useful)**
   1. Verbal information from the doctor

0 1 2 3 4 5 6 7 8 9 10

- 1. Verbal information from the nurses

0 1 2 3 4 5 6 7 8 9 10

- 1. Written information leaflet provided on PGS

0 1 2 3 4 5 6 7 8 9 10

1. **Why did you choose to have PGS done? (please tick where applicable)**

Repetitive failed embryo transfers/implantations (two or more)

Recurrent miscarriage (three or more)

Advanced maternal age

Previous fetus/baby affected by chromosomal abnormality

Personal request

1. **How many IVF cycles did you need to have a suitable embryo for transfer using PGS?**

One  Two  Three  Three  Four  Five  Six or more

**Please rate the extent you agree or disagree with the following statements:**

1. **I believe PGS is reliable and gives consistently accurate results regarding the chromosomal make-up of an embryo**

Strongly agree  Agree  Neither agree nor disagree

Disagree  Strongly disagree

1. **I believe future technological advances will lead to a significant improvement in IVF outcome**

Strongly agree  Agree  Neither agree nor disagree

Disagree  Strongly disagree

1. **What was your understanding about your chance of achieving a livebirth once a suitable embryo was transferred?**

0-20%  20-40%  40-60%  60-80%  80-100%  Unsure

1. **What was your understanding about your chance of a miscarriage once a suitable embryo was transferred?**

0-20%  20-40%  40-60%  60-80%  80-100%  Unsure

1. **At the time of doing PGS, did you think that PGS would guarantee you to have a livebirth free from chromosomal abnormalities?**

Yes  No  Unsure

1. **At the time of doing PGS, was it explained to you that if you fall pregnant, routine pre-natal screening in pregnancy is still recommended to confirm the PGS result of a genetically normal embryo?**

Yes  No  Unsure

1. **If your own fertility treatment was successful, do you plan to tell any children from your treatment that you used IVF with PGS?**

Yes  No  Unsure  N/A (I did not become pregnant)

**Thank you for taking the time to fill out this questionnaire.**
